# Moment Dynamics of Zirconia Particle Formation for Optimizing Particle Size Distribution

**DOI:** 10.3390/nano9030333

**Published:** 2019-03-02

**Authors:** Wolfgang Halter, Rahel Eisele, Dirk Rothenstein, Joachim Bill, Frank Allgöwer

**Affiliations:** 1 Institute for Systems Theory and Automatic Control, University of Stuttgart, 70569 Stuttgart, Germany; frank.allgower@ist.uni-stuttgart.de; 2 Institute for Materials Science, University of Stuttgart, 70569 Stuttgart, Germany; raheleisele@gmx.de (R.E.); dirk.rothenstein@imw.uni-stuttgart.de (D.R.); joachim.bill@imw.uni-stuttgart.de (J.B.)

**Keywords:** particle formation, moment dynamics, parameter identification, parameter optimization, zirconia-based material

## Abstract

We study the particle formation process of Zirconia (${\mathrm{ZrO}}_{2}$)-based material. With a model-based description of the particle formation process we aim for identifying the main growth mechanisms for different process parameters. After the introduction of a population balance based mathematical model, we derive the moment dynamics of the particle size distribution and compare the model to experimental data. From the fitted model we conclude that growth by molecular addition of Zr-tetramers or Zr-oligomers to growing particles as well as size-independent particle agglomeration takes place. For the purpose of depositing zirconia-based material (ZrbM) on a substrate, we determine the optimal process parameters such that the mineralization solution contains preferably a large number of nanoscaled particles leading to a fast and effective deposition on the substrate. Besides the deposition of homogeneous films, this also enables mineralization of nanostructured templates in a bioinspired mineralization process. The developed model is also transferable to other mineralization systems where particle growth occurs through addition of small molecular species or particle agglomeration. This offers the possibility for a fast determination of process parameters leading to an efficient film formation without carrying out extensive experimental investigations.

## 1. Introduction

Zirconia (${\mathrm{ZrO}}_{2}$) is an oxide material with versatile properties such as chemical, thermal and mechanical resistance along with a high refractive index, beneficial electrical properties and bio-compatibility [1,2,3,4]. These properties enable various application possibilities, such as the use as thermal barrier coating or corrosion protection [5,6], as a filler for nanocomposites [7,8], as photocatalyst [9] or catalyst for ${\mathrm{CO}}_{2}$ methanation [10]. It can be used for optical, electrical and dental applications, as implant material, for example for a femoral head of a hip implant and for enhancing the performance of Li-Ion batteries [11,12,13,14,15,16].

For producing such advanced ceramics, nanosized particles that are stable against agglomeration are required. These particles enable the production of homogeneous ceramics in terms of density and surface roughness. The latter is especially true for thin layers. As a low cost method for the production of ${\mathrm{ZrO}}_{2}$-based particles, Hu et al. [17] describe a thermohydrolytic method using inorganic metal salts, like ${\mathrm{ZrOCl}}_{2}\;\cdot \;8H_{2}O$, as precursor. Applying an alcohol-water solvent mixture leads to enhanced nucleation and growth rates of the particles due to the low dielectric constant of alcohol and the alcohol-water mixture, allowing a reduced process temperature and resulting in an even cheaper method for particle formation [6,17,18,19,20,21].

The production of nanostructured ceramics using conventional manufacturing methods, such as chemical vapour deposition or physical vapour deposition processes, sputtering techniques or thermohydrolytic methods [22], is still challenging. In order to overcome this challenge, nature is taken as a model. In nature, precise nanostructures can be produced under ambient conditions [23,24]. Examples include living organisms such as mussels [25] or glass sponges [26,27]. These living organisms use organic templates to control material formation [28,29] and the type of material formation is known as “biologically controlled biomineralization”. Transferring this manufacturing principle to the production of technically interesting materials is a current topic in research referred to as “bioinspired mineralization” [30,31,32]. In general, material formation on a template can take place through two types of nucleation: homogeneous and heterogeneous nucleation [33]. In case of homogeneous nucleation, nucleation takes place in solution. Particles, which are formed from the nuclei, are deposited on the template. In case of heterogeneous nucleation, nucleation takes place directly on the template. In such a template-controlled material formation process, either by heterogeneous or homogeneous nucleation, it is important to ensure a large interaction area between the inorganic material and the template. This enables a large template influence on material formation. For the heterogeneous nucleation by attachment of small molecular species on the template in most cases a large interaction area is given by the fact that the template is large compared to the molecular species. For homogeneous nucleation a large interaction area is ensured by the production of small particles in the lower nanometer range. In this case, it is important that the formed nanoparticles are stable against agglomeration, which ensures the formation of homogeneous films. With both types of nucleation, care must also be taken to ensure that the template remains chemically and thermally stable with regard to the pH value and the respective temperature. In this work we focus on the formation of nanosized particles that are stable against agglomeration. In order to be able to identify mineralization conditions that meet this requirement without extensive experimental investigations, a mathematical model is developed that describes the process of particle formation and the process of particle growth including particle agglomeration. The model is based on experimental investigations of particle formation in a ${\mathrm{ZrOCl}}_{2}$ solution in an ethanol-water solvent. The influence of different solution- and process parameters on particle formation and particle growth is investigated.

Previous detailed studies to describe the influence of different solution- and process parameters on the particle formation and morphology were mainly performed in isopropanol-water solvent mixtures [17,19,20]. However, Moon et al. [18] observed a different behavior of particle formation for other alcohol-water solvent mixtures. This was attributed to different dielectric constants of the respective alcohols. In an ethanol-water solvent mixture, very fine particles were formed in the lower nanometer range. Other alcohols such as 1-propanol, 2-propanol or tert-butyl alcohol led to the formation of larger particles. Therefore, the solvent mixture ethanol-water was chosen in the present study in order to obtain nanosized particles for the targeted mineralization of templates. With the developed mathematical model of the particle formation process, the main mechanism of particle growth at different system parameters can be identified. Further, with such a model at hand, the system parameters are optimized to maximize the number of nanoparticles for the mineralization of thin, homogeneous films.

This is the first time a mathematical model has been developed to describe the particle formation process, identify the main mechanism for particle growth, and enable a model based optimization of process parameters. This model, developed for a specific mineralization system (${\mathrm{ZrOCl}}_{2}$ in ethanol-water solvent mixture (80 vol.% ethanol)), can be transferred to other mineralization systems showing similar particle growth mechanisms, i.e., particle growth by molecular addition of small species formed from inorganic metal salts and particle growth by particle agglomeration. Thus, in a fast and simple way, process parameters for other mineralization systems, which lead to the formation of a high number of nanoparticles, can be identified allowing for a precise mineralization of templates or the mineralization of homogeneous layers.

After the introduction of the physico-chemical process of particle formation, the general population balance model of the particle formation process is introduced. Subsequently, this model is simplified such that only the dynamics of the moments are considered. We then use the moment model and acquired experimental data to identify the system parameters and, in particular, the dependencies of these parameters on process variables such as temperature and precursor solution concentration. Given these dependencies we finally derive the optimal experimental conditions which lead to a maximum number of nanoparticles.

## 2. Materials and Methods

### 2.1. Materials

For the mineralization solution ${\mathrm{ZrOCl}}_{2}\;\cdot \;8H_{2}O$ (Sigma-Aldrich, Steinheim, Germany, 98% purity) was used (20mM, 30mM and 50mM). As solvent a mixture of ${\mathrm{ddH}}_{2}O$ (18.1MΩcm) and ethanol (Roth, Karlsruhe, Germany, ≥99.8%, p.a., purity) with 80 vol.% ethanol was chosen. For zetapotential measurements of mineralized particles in 10mM sodium chloride (Merck, Darmstadt, Germany) the pH value was adjusted using hydrochloric acid (Roth, fuming 37%) and sodium hydroxide (Merck).

### 2.2. Methods

#### 2.2.1. Dynamic Light Scattering

Particle sizes in the mineralization solution were measured by dynamic light scattering (DLS) on a ZETASIZER 3000 HSA (Malvern Instruments, Herrenberg, Germany) with a He-Ne laser (λ=633nm). The scattered light was detected at an angle of $90^{\circ }$. The mineralization solutions were analyzed in a 1.5mL UV-disposable cuvette (BRAND, Wertheim, Germany). The temperature was kept constant by a temperature-controlled cuvette holder. A total of 30 measuring points were recorded at intervals of 128s or 256s (alternating). Each measuring point represented the mean value of 10 individual measurements. The mean particle size was calculated with the software PCS v1.52 (Malvern Instruments). This required the refractive index of the particles and the solvent as well as the viscosity of the solvent. For the solvent, water ethanol (80 vol.% ethanol), the values of pure ethanol were used. At $25{\;}^{\circ }C$, ethanol has a refractive index of 1.36 and a viscosity of 1.1mPas. For the particles, the refractive index of ${\mathrm{ZrO}}_{2}$ was used with a real part of 2.16 and an imaginary part of 0.1. Three independent experiments were performed per sample.

#### 2.2.2. Optical Emission Spectrometry with Inductively Coupled Plasma

Using optical emission spectrometry with inductively coupled plasma (inductively coupled plasma optical emission spectrometry, ICP-OES), the zirconium consumption during mineralization at $25{\;}^{\circ }C$, $40{\;}^{\circ }C$ and $60{\;}^{\circ }C$ was determined. The mineralization solution was split into 1mL aliquots. For mineralization at $40{\;}^{\circ }C$ or $60{\;}^{\circ }C$, the solutions were heated in an oil bath. The temperature was controlled with a heatable magnetic stirrer through a thermostat. After 0, 10, 50 and 90min the mineralization was stopped by transferring the solution on ice. The mineralization product was removed by centrifugation (10min, 20,817× *g*). The Zr-content of the supernatant was determined with an ICP-OES spectrometer (Spectro Ciros, Spectro Analytical Instruments, Kleve, Germany). The sample aerosol was injected into an argon plasma with a temperature of 8000–10,000 K. Three independent experiments were performed per sample. An aqueous ${\mathrm{ZrOCl}}_{2}$ solution, which does not form a precipitate at room temperature (could be shown by DLS measurements and ICP-OES measurements), served as a reference.

#### 2.2.3. Liquid Pycnometry

The density of mineralization products (ZrbM) was determined by liquid pycnometry. ZrbM was mineralized for 1.5h at $40{\;}^{\circ }C$ or for 4 months at $25{\;}^{\circ }C$. It was then centrifuged (10min, 20,817× *g*) and dried overnight at room temperature. A glass pycnometer with a volume of 24.944mL was used to determine the density. An ethanol-water mixture (80 vol.% ethanol) corresponding to the solvent in the mineralization solution was used as liquid. The density of ZrbM was determined by the displaced volume of ethanol-water according to

(1) $$\rho_{ZrbM}=\frac{\left(m_{2}-m_{0}\right)}{\left(m_{1}-m_{0}\right)-\left(m_{3}-m_{2}\right)}\;\cdot \;\rho_{Eth/W}.$$

The mass of the empty pycnometer ($m_{0}$) and the masses of the pycnometer filled with the ethanol-water mixture ($m_{1}$), filled with ZrbM ($m_{2}$) and filled with ZrbM and the ethanol-water mixture ($m_{3}$) were used. The respective masses were determined at $25{\;}^{\circ }C$. The density of ethanol-water $\rho_{Eth/W}$ at $25{\;}^{\circ }C$ was also determined by pycnometry:

(2) $$\rho_{Eth/W}=\frac{\left(m_{1}-m_{0}\right)}{V_{p}},$$

where $V_{p}$ represents the volume of the pycnometer.

#### 2.2.4. Zeta Potential Measurements

The zeta potential of mineralized particles from ZrbM was determined with a Zetasizer Nano (Malvern Instruments) in folded capillary cells (DTS1070). Mineralized particles of ZrbM were centrifuged from the mineralization solution (10min, 20,817×g). After resuspension in an ethanol-water mixture (80 vol.% ethanol) a further centrifugation step of 10min at 20,817×g followed. For the zeta potential measurement the particles were resuspended in 10mMNaCl at pH values in the range of 2.4 to 9.8. The pH value was adjusted by adding hydrochloric acid or sodium hydroxide. For the zeta potential measurements at least 12 single measurements were performed at $25{\;}^{\circ }C$. Two independent samples were characterized for each pH value. The Smoluchowsky approach was used for evaluation [34].

## 3. Results

In this section we present the population balance based model, the results of the parameter fitting and the optimization of process parameters.

### 3.1. Mineralization of ZrbM

Crystalline ${\mathrm{ZrOCl}}_{2}\;\cdot \;8H_{2}O$ is made of ${\left[{\mathrm{Zr}}_{4}{\left(\mathrm{OH}\right)}_{8}{\left(H_{2}O\right)}_{16}\right]}^{8+}$ tetramers, which also exist after the dissolution of the salt in water [35,36]. Coordinated water molecules can be deprotonated resulting in ${\left[{\mathrm{Zr}}_{4}{\left(\mathrm{OH}\right)}_{8+x}{\left(H_{2}O\right)}_{16-x}\right]}^{(8-x)+}$ with a size of 0.8nm [17]. By olation reactions between hydroxy groups and coordinated water molecules, oligomerization of the tetrameric complex takes place. Initially, there is an equilibrium between the tetrameric and octameric complex (1.2nm), like described by Singhal et al. [37] and Cölfen et al. [38], i.e.,

(3) $${\left[{\mathrm{Zr}}_{4}{\left(\mathrm{OH}\right)}_{8}{\left(H_{2}O\right)}_{16}{\mathrm{Cl}}_{6}\right]}^{2+}⇌\;\left[{\mathrm{Zr}}_{8}{\left(\mathrm{OH}\right)}_{20}{\left(H_{2}O\right)}_{24}{\mathrm{Cl}}_{12}\right]+4H^{+}+4H_{2}O.$$

At elevated temperatures, further olation and oxolation reactions lead to higher polymeric species with a lower solubility than the smaller ones. Finally, oversaturation of the higher polymeric species causes nucleation. Primary particles with size <5nm are formed [17]. Particle growth may occur either through molecular addition of small Zr-species like Zr-tetramers or Zr-oligomers or by particle agglomeration to secondary particles. In this work we use an ethanol-water solvent mixture according to Moon et al. [18], who synthesized small, soft-agglomerated particles. In this reaction ZrO${}_{2}$·2H${}_{2}$O is synthesized (Moon et al. [18]). This zirconia-based material (ZrbM) has a molar mass of $M=159\times 10^{-3}\;kg/\mathrm{mol}$ (calculated) and a density of $\rho =1.2\pm 0.2\;g/cm^{3}$ (determined by pycnometry). According to TGA and DSC measurements (not shown) the crystallization of the amorphous ZrbM to tetragonal ${\mathrm{ZrO}}_{2}$ takes place at about $450{\;}^{\circ }C$. At higher temperatures of about $900{\;}^{\circ }C$ the tetragonal phase transforms partially into the monoclinic phase.

**Figure 1:**
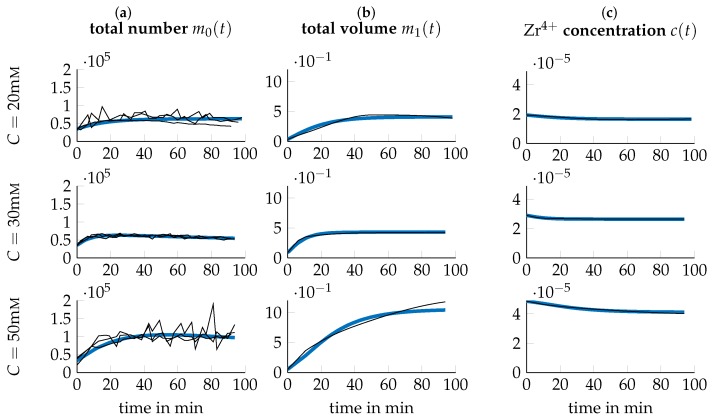
Data (black thin lines) and simulation (blue thick lines) for $T=25{\;}^{\circ }C$ and different precursor concentrations *C*. The time-series of the data are calculated according to Section 3.3 from the temporal evolutions of the average particle diameter of the particle population obtained from a single DLS run and the average concentration of ${\mathrm{Zr}}^{4+}$ obtained from three independent ICP measurements. The three black lines correspond to three independent DLS runs.

**Figure 2:**
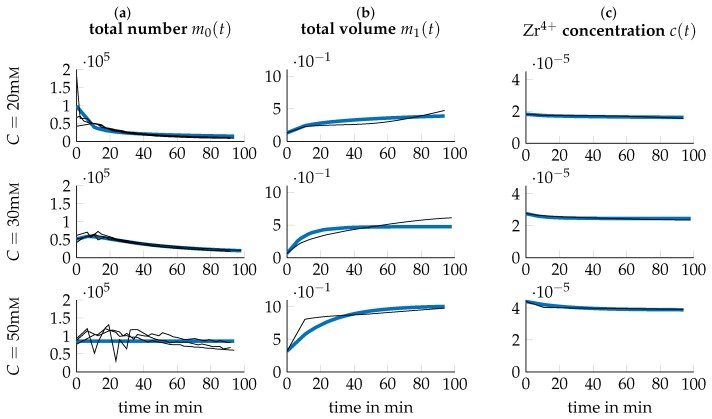
Data (black thin lines) and simulation (blue thick lines) for $T=40{\;}^{\circ }C$ and different precursor concentrations *C*. Data obtained like in Figure 1.

**Figure 3:**
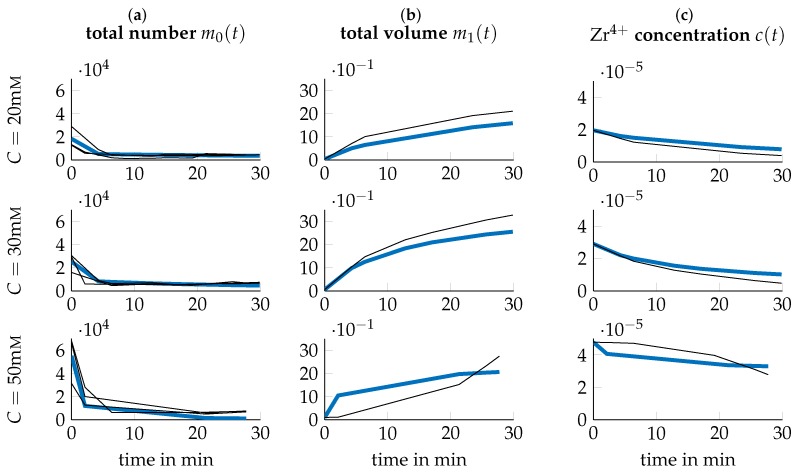
Data (black thin lines) and simulation (blue thick lines) for $T=60{\;}^{\circ }C$ and different precursor concentrations *C*. Data obtained like in Figure 1.

Experimental data of the system under study was collected by conducting a series of *I* number of experiments under different process parameters, i.e., varying temperature *T* and precursor concentration *C*. We thus introduce the *I* experimental conditions as the tupels $\xi_{i}=\left\{T_{i},C_{i}\right\}$, i∈[1,I] where $T_{i}$ and $C_{i}$ stand for the temperature *T* and precursor concentration *C* of experimental condition *i* respectively. For condition *i*, the temporal evolution of the concentration of ${\mathrm{Zr}}^{4+}$ in soluble Zr-species like Zr-tetramers ($c(t,\xi_{i})$, [c]=mol/mL) is acquired using inductively coupled plasma optical emission spectrometry (ICP-OES) measurements. Mean values of these measurements are depicted as black lines in columns (c) of Figure 1, Figure 2 and Figure 3. Further, the average particle diameter ($Z(t,\xi_{i})$, [Z]=mm) is determined using DLS measurements.

### 3.2. Model

For the mathematical model of particle formation, we restricted ourself to modeling the mechanisms of nucleation, size-dependent agglomeration and growth by molecular addition of Zr-tetramers or -oligomers. We considered a system with constant reaction volume and temperature. According to Randolph and Larson [39], the population balance equation for such a system is then given by

(4) $$\begin{array}{c} \frac{\partial n(t,V)}{\partial t}+G\frac{\partial (n(t,V))}{\partial V}=B\left(V\right)-D\left(V\right), \end{array}$$

where n(t,V) stands for the number density at time *t* dependent on the characteristic particle volume *V*, *G* denotes the particle growth rate and B(V) and D(V) are volume dependent birth and death rates, respectively. We further assumed that the growth rate *G* is size independent, and that the birth rate B(V) is determined by the processes of nucleation and agglomeration, while the death rate D(V) is determined by agglomeration alone. Note that, in contrast to classical Lotka–Volterra models, the birth and death rates will not be simple linear or nonlinear functions of the current number of particles but that they rather implicitly depend on the current continuous distribution of particle number over particle volume, leading ultimately to an integro-differential equation. According to Worlitschek and Mazzotti [40], the growth rate is proportional to the level of supersaturation and obeys different laws for unsaturated conditions. As we are studying particle formation, we assume in the remainder that the solution is always in supersaturation and thus define G=a·△c, where *a* is an unknown parameter to be determined and $△c=c\left(t\right)-c_{s}$ is the level of supersaturation, viz. the difference between *c*, the concentration of ${\mathrm{Zr}}^{4+}$-ions at time *t* in the solution and the saturation concentration $c_{s}$, which is potentially temperature dependent.

Due to nucleation and agglomeration, following the results of Worlitschek and Mazzotti [40] and Hounslow et al. [41], the birth rate of new particles with volume *V* is given by

(5) $$\begin{array}{cc} B(V) & =\left\{\begin{array}{cc} B_{0} & V=0\\ \frac{1}{2}\int_{0}^{V}\beta \left(V-\epsilon ,\epsilon \right)\;\cdot \;n\left(V-\epsilon \right)\;\cdot \;n\left(\epsilon \right)d\epsilon & V>0 \end{array}\right. \end{array}$$

(6) $$\begin{array}{c} =B_{0}\delta \left(V\right)+\frac{1}{2}\int_{0}^{V}\beta \left(V-\epsilon ,\epsilon \right)\;\cdot \;n\left(V-\epsilon \right)\;\cdot \;n\left(\epsilon \right)d\epsilon , \end{array}$$

with $B_{0}=d\;\cdot \;△c$ the nucleation rate of newly formed particles, which are of theoretical size zero, and the agglomeration kernel β(V−ϵ,ϵ), which describes the probability that two particles with volume V−ϵ and ϵ collide and successfully form a new particle of volume *V*. Therein, δ(V) denotes the standard Dirac delta distribution in *V*, defined such that

(7) $$\begin{array}{c} \int_{-\infty }^{\infty }\delta \left(V\right)=1, \end{array}$$

and in particular with an arbitrary function *f*

(8) $$\begin{array}{c} \int_{-\infty }^{\infty }f\left(V\right)\delta \left(V\right)=f\left(0\right). \end{array}$$

The death rate respectively is given by

(9) $$\begin{array}{c} D\left(V\right)=n\left(V\right)\;\cdot \;\int_{0}^{\infty }\beta \left(V,\epsilon \right)\;\cdot \;n\left(\epsilon \right)d\epsilon . \end{array}$$

For the choice of an appropriate agglomeration kernel, different approaches are possible, dependent on the nature of the system [42]. In our case, a two stage kernel as discussed in Roy et al. [43] of the form

(10) $$\begin{array}{c} \beta \left(V,\epsilon \right)=\beta_{1}+\beta_{2}V\epsilon , \end{array}$$

was chosen to account for both size-independent and size-dependent agglomeration.

For direct simulation of Equation (4), approaches like the method of characteristics or the method of lines are applicable [44,45]. This leads to the challenge of determining an approximation of $\frac{\partial n}{\partial V}$, as well as solving the convolution integral in Equation (6). Also, the change in concentration of the surrounding medium needs to be simulated simultaneously. This in general leads to a model description which necessitates a comparably high computational effort for simulation. As it is necessary to identify several unknown model parameters, long simulation times are undesired since this would slow down the identification process critically. Another approach to solving the PDE Equation (4) is by application of an integral transformation. In the remainder of this section, we therefore simplified Equation (4) by deducing the moment dynamics of the system and thus arriving at an ordinary differential equation (ODE) description of the dynamics which is much simpler and faster to simulate. Note that also Fourier or Laplace transformations could be applied, however, using the moment dynamics has the advantage of conserving a physical interpretation of the transformed variables.

First, we defined the *i*-th moment as

(11) $$\begin{array}{c} m_{i}:=\int_{0}^{\infty }V^{i}n\left(V\right)dV, \end{array}$$

and realized that the zeroth moment gives the total number of particles and the first moment gives total volume of solid particles, two entities for which we later show that measurement data can be obtained. Under the assumption of a closed system, the amount of ${\mathrm{Zr}}^{4+}$ which is missing from the solution is equal to the amount of solid ZrbM and we, thus, find a relation between the concentration of solute and the first moment as

(12) $$\begin{array}{cc} c & =c_{0}-\frac{\rho }{M}m_{1}. \end{array}$$

This in turn means that both the growth rate *G* and nucleation rate $B_{0}$ are functions of $m_{1}$. However, for the sake of simplicity that we adhered to the notation as it was introduced for the remainder of this work.

Taking the temporal derivative of Equation (11) and using (4) we arrived at

(13) $$\begin{array}{cc} {\dot{m}}_{i} & =\int_{0}^{\infty }V^{i}\left(B\left(V\right)-D\left(V\right)-G\frac{\partial (n(t,V))}{\partial V}\right)dV, \end{array}$$

and for the integration in parts, we defined ${\dot{m}}_{i}=I_{1}+I_{2}+I_{3}$ with

(14) $$\begin{array}{cc} I_{1} & =\int_{0}^{\infty }V^{i}B\left(V\right)dV \end{array}$$

(15) $$\begin{array}{cc} I_{2} & =-\int_{0}^{\infty }V^{i}D\left(V\right)dV \end{array}$$

(16) $$\begin{array}{cc} I_{3} & =-G\int_{0}^{\infty }V^{i}\frac{\partial (n(t,V))}{\partial V}dV. \end{array}$$

For simplifying Equation (14), we first realized that according to the identity given in Equation (8),

(17) $$\begin{array}{c} \int_{0}^{\infty }V^{i}B_{0}\delta \left(V\right)dV=0^{i}B_{0} \end{array}$$

holds. Next, we rearranged the remaining integral and due to the property that n(V−ϵ)=0, ∀ϵ≥V a change of integration boundaries is possible, s.t.

(18) $$\begin{array}{c} \int_{0}^{\infty }V^{i}\frac{1}{2}\int_{0}^{V}\beta \left(V-\epsilon ,\epsilon \right)\;\cdot \;n\left(V-\epsilon \right)\;\cdot \;n\left(\epsilon \right)d\epsilon \;dV\\ =\frac{1}{2}\int_{0}^{\infty }\int_{0}^{\infty }V^{i}\beta \left(V-\epsilon ,\epsilon \right)\;\cdot \;n\left(V-\epsilon \right)\;\cdot \;n\left(\epsilon \right)d\epsilon \;dV. \end{array}$$

We now made a change of the integration variable according to u=V−ϵ, du=dV and arrive at

(19) $$\begin{array}{cc} I_{1} & =0^{i}B_{0}+\frac{1}{2}\int_{0}^{\infty }\int_{0}^{\infty }{\left(u+\epsilon \right)}^{i}\left(\beta_{1}+\beta_{2}u\epsilon \right)n\left(u\right)n\left(\epsilon \right)d\epsilon \;du \end{array}$$

(20) =0iB0+12∑k=0iikβ1mi−kmk+β2mi−k+1mk+1.

where we expanded ${\left(u+\epsilon \right)}^{i}$ with the binomial theorem and rearranged some terms of the integrand.

The simplification of $I_{2}$ is straightforward and with some simple rearrangements we got

(21) $$\begin{array}{cc} I_{2} & =-\beta_{1}m_{i}m_{0}-\beta_{2}m_{i+1}m_{1}. \end{array}$$

It remained to simplify $I_{3}$, which can be achieved by partial integration and the assumption that n(0)=n(∞)=0, arriving at

(22) $$\begin{array}{cc} I_{3} & =iGm_{i-1}. \end{array}$$

We noted that these results in general imply that the dynamics of the *i*-th moment depend on moments up to the degree of i+1, thus yielding a system of ODEs of infinite dimension. However, if we consider the zeroth and first moments only, it turns out that $I_{1}$ and $I_{2}$ just cancel out in the equation for ${\dot{m}}_{1}$, thus leading to the closed moment dynamics

(23) $$\begin{array}{cc} {\dot{m}}_{0} & =B_{0}-\frac{1}{2}\left(\beta_{1}m_{0}^{2}+\beta_{2}m_{1}^{2}\right) \end{array}$$

(24) $$\begin{array}{cc} {\dot{m}}_{1} & =Gm_{0} \end{array}$$

### 3.3. Data Transformation

As mentioned before, our goal was to identify the main growth mechanism of particle formation and further find optimal process parameters such that the particle formation yields a large number of small particles. We therefore developed a simple mathematical model of particle formation for which we now need to identify certain process parameters. Due to the fact that the model is given in terms of the moments, i.e., the total amount $N_{T}\left(t,\xi_{i}\right)$ and volume $V_{T}\left(t,\xi_{i}\right)$ of particles per unit volume of solution, it is necessary to transform the obtained time series measurements of the concentration of ${\mathrm{Zr}}^{4+}$ in soluble Zr-species like Zr-tetramers ($c(t,\xi_{i})$, [c]=mol/mL) and the average particle diameter ($Z(t,\xi_{i})$, [Z]=mm) into respective quantities.

According to Equation (12) we find $V_{T}$ at time *t* as

(25) $$\begin{array}{c} V_{T}\left(t,\xi_{i}\right)=\left(c_{0}-c\left(t,\xi_{i}\right)\right)\frac{M}{\rho },\;\left[V_{T}\right]=mm^{3}/mL. \end{array}$$

Note that due to the design of the experiment, we assumed that $c_{0}=c\left(0,\xi_{i}\right)=C_{i}$. It remained to derive $N_{T}\left(t,\xi_{i}\right)$ from $Z(t,\xi_{i})$. Therefore, we introduce the quantities $D_{T}$ and $V_{p}$, the total diameter of particles per unit volume and the particular (average) volume of one particle respectively as

(26) $$\begin{array}{cc} D_{T} & =Z\;\cdot \;N_{T} \end{array}$$

(27) $$\begin{array}{cc} V_{p} & =\frac{\pi }{6}Z^{3}. \end{array}$$

Solving Equation (26) for *Z* and substituting it in Equation (27) we arrived at

(28) $$\begin{array}{cc} V_{p} & =\frac{\pi }{6}{\left(\frac{D_{T}}{N_{T}}\right)}^{3}. \end{array}$$

Now since $V_{T}=\frac{\pi }{6}D_{T}^{3}$ also needs to hold, we found

(29) $$\begin{array}{cc} N_{T}\left(t,\xi_{i}\right) & ={\left(\frac{V_{T}\left(t,\xi_{i}\right)}{V_{p}\left(t,\xi_{i}\right)}\right)}^{\frac{1}{3}}={\left(\frac{V_{T}\left(t,\xi_{i}\right)}{\frac{\pi }{6}Z{\left(t,\xi_{i}\right)}^{3}}\right)}^{\frac{1}{3}}, \end{array}$$

with $[N_{T}]={mL}^{-1}$, concluding the necessary transformations. The resulting quantities are depicted in Figure 1, Figure 2 and Figure 3 respectively. Note that for $m_{0}\left(t\right)=N_{T}\left(t\right)$, all three triplicates are plotted while for $m_{1}\left(t\right)=V_{T}\left(t\right)$ and c(t) only the mean over the ICP-OES experiments are included.

### 3.4. Parameter Identification

For fitting the model to the transformed data, particle growth rate *a*, particle nucleation rate *d*, saturation concentration $c_{s}$, size-independent agglomeration rate $\beta_{1}$ and size-dependent agglomeration rate $\beta_{2}$ had to be identified for each experimental condition. Ultimately, the dependence of these mechanistic parameters on the changing conditions *T* and *C* shall be identified. We therefore introduced the mechanistic parameter vector $\phi ={\left[a,d,c_{s},\beta_{1},\beta_{2}\right]}^{⊤}$ and assumed a second order polynomial dependency of the mechanistic parameters ϕ on the process parameters *T* and *C*, i.e.,

(30) $$\begin{array}{c} \phi_{k}=\alpha_{k}\;\cdot \;\left(p_{k1}T^{2}+p_{k2}TC+p_{k3}C^{2}+p_{k4}T+p_{k5}C+p_{k6}\right) \end{array}$$

for the *k*-th parameter, with $\alpha_{k}$ a manually chosen weighting parameter to regularize the later following optimization problem and $p_{kj}$ denoting the decision variables which we collect to one vector, viz. $p=[p_{kj}]$, $k\in \left[1,5\right],\;j\in \left[1,6\right],\;p\in R^{30}$. Let now $m_{0}\left(t_{m},\xi_{i},p\right)$ and $m_{1}\left(t_{m},\xi_{i},p\right)$ be the solutions of Equations (23) and (24) at the time $t_{m}$ for experimental condition $\xi_{i}$ under the choice of parameters *p* respectively. We defined the objective function

(31) $$\begin{array}{cc} J(p) & =\sum_{i=1}^{I}\sqrt{\sum_{m}\left[{\left(R_{0}\left(m,i,p\right)\right)}^{2}+{\left(R_{1}\left(m,i,p\right)\right)}^{2}\right]}, \end{array}$$

with the residuals

(32) $$\begin{array}{cc} R_{0}\left(m,i,p\right) & =\frac{N_{T}\left(t_{m},\xi_{i}\right)-m_{0}\left(t_{m},\xi_{i},p\right)}{\mathrm{Md}(N_{T}\left(\;\cdot \;,\xi_{i}\right))} \end{array}$$

(33) $$\begin{array}{cc} R_{1}\left(m,i,p\right) & =\frac{V_{T}\left(t_{m},\xi_{i}\right)-m_{1}\left(t_{m},\xi_{i},p\right)}{\mathrm{Md}(V_{T}\left(\;\cdot \;,\xi_{i}\right))}, \end{array}$$

and Md(x) denoting the median of sample *x*, which is used to turn the acquired data into dimensionless normalized data. The model fitting now reduces to the following optimization problem:

Find $\hat{p}$ such that

(34) $$\begin{array}{c} J\left(\hat{p}\right)=\min_{p}J\left(p\right). \end{array}$$

**Figure 4:**
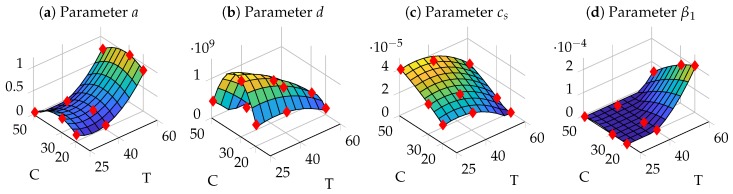
Polynomial dependencies of mechanistic parameters on temperature (T) and precursor concentration (C). Red diamonds indicate the parameter values at the provided experimental conditions.

We solved this optimization problem with the patternsearch algorithm implemented in MATLAB and the resulting polynomial functions are depicted in Figure 4. Therein, red diamonds indicate the parameter values for the provided experimental data. The dependence of $\beta_{2}$ is not depicted as the optimization suggested that $\beta_{2}=0\;\forall \xi_{i}$, i.e., no size-dependent agglomeration takes place.

Interestingly, the saturation concentration $c_{s}$ increased linearly with increasing precursor concentration (Figure 4c), which at first seems to be counter intuitive. However, higher precursor concentrations also lead to a lower pH of the solution which according to Equation (3) in turn favors tetramers or oligomers, as those are more stable under these conditions. In comparison to large Zr-species like polymers, small Zr-species like tetramers and oligomers reveal a higher solubility. Thus, the saturation concentration $c_{s}$ of tetramers or oligomers is higher [46]. With increasing temperature the value of $c_{s}$ decreases (Figure 4c). This may be due to a decrease of the dielectric constant ϵ with increasing temperature as reported [20]. Nucleation of a solid phase from a solution of charged Zr-species, like charged Zr-tetramers, takes place by formation of a neutral complex by addition of chloride counterions [47]. The following applies to particle formation in a solution supersaturated with Zr-species [48,49]:

(35) $$\begin{array}{c} c_{s}\approx \exp\left(-\frac{z_{+}z_{-}e^{2}}{4\pi \epsilon_{0}\epsilon k_{B}T\left(r_{+}+r_{-}\right)}\right), \end{array}$$

where $z_{+}$ and $z_{-}$ are the ionic charges, $r_{+}$ and $r_{-}$ are the ionic radii, *e* is the elementary charge, $\epsilon_{0}$ is the permittivity of the vacuum and $k_{B}$ the Boltzmann constant. According to Equation (35), $c_{s}$ decreases with decreasing ϵ and, thus, with increasing *T*. Additionally, as Hu et al. [17] indicate, the rates of hydrolysis and condensation reactions of Zr-tetramers increase with higher temperature, leading to oligomers which have a decreased solubility (smaller $c_{s}$) compared to smaller ones.

Concerning agglomeration, a size independent agglomeration ($\beta_{1}$) can be observed for high temperatures and low precursor concentrations. The particle agglomeration can be influenced by attractive or repulsive interactions between the particles [50,51]. Especially repulsive electrostatic interactions [52] as well as attracting Van der Waals interactions [50] play an important role. For small particle spacings, Born repulsion [51,53,54] also acts due to the overlapping of the electron clouds. In the mineralization system considered here, the particles are suspended in a ${\mathrm{ZrOCl}}_{2}$ solution with an ethanol-water solvent mixture (80 vol.% ethanol). Calculations of the interaction energy between particles of the same size regarding electrostatic and van der Waals interactions as well as Born repulsion according to literature [50,51,52,53,54] resulted in attractive interactions between the particles at particle spacings between 1.5nm and 3.5nm (calculation not shown). For these calculations a particle diameter of 10nm, a zeta potential of 40mV (pH 2.4), ${\mathrm{ZrOCl}}_{2}$ concentrations between 20mM and 50mM and temperatures between 25${\;}^{\circ }C$ and 60${\;}^{\circ }C$ were used. However, these attractive interaction energies were at least 32 times smaller than the thermal energy. As a result, thermal particle movement counteracts agglomeration. However, this also means that other factors must contribute to agglomeration. A chemical bond between particles can be formed e.g., by condensation reactions between surface hydroxy groups. This condensation reaction is influenced both by the pH value and by the temperature [17,37,38]. Thus, the negative correlation of the agglomeration with concentration (Figure 4d) may again be caused by the changing pH of the solution, where a higher pH (lower ${\mathrm{ZrOCl}}_{2}$ concentration) according to Equation (3) favors the condensation reaction. Higher temperatures also promote the condensation reaction [17,37,38] and, thus, lead to increased agglomeration (Figure 4d).

The dependencies of the growth rate *a* and the nucleation rate *d* on the process parameters are mainly a result of the discussed mechanisms and in particular determined by the parameter values of $c_{s}$ and $\beta_{1}$. This leads to a negative quadratic influence of the precursor concentration, leading to a saddle-like manifold for parameter *a* and a parabola for parameter *d*. This also implies that, if extrapolating the experimental conditions beyond the acquired data, both values change their sign from a positive to a negative value eventually, leading to a physically meaningless behavior. For the phenomenological description of these parameters we therefore set the lower limit of these values to zero and propose for future work to collect more data in areas where zero growth or nucleation is predicted.

Both the acquired data as well as the simulation of the model at the found $\hat{p}$ is depicted in Figure 1, Figure 2 and Figure 3 for all tested experimental conditions. For a process temperature of $25{\;}^{\circ }C$, depicted in Figure 1, data and simulations concur extensively for all tested precursor concentrations. The same applies to the other temperatures. However, there are two exceptions, viz. for $T=40{\;}^{\circ }C,\;C=20\;mM$ and $T=60{\;}^{\circ }C,\;C=50\;mM$, where the plot of the precursor concentration over time has a slightly concave shape. As this qualitatively differs significantly from the other experimental conditions, where the precursor concentration has a convex shape, further replicates should be considered to verify this observation. However, DLS measurements were not possible for samples at $T=60{\;}^{\circ }C$ and t≥30min, since particle concentration was already too high.

### 3.5. Optimization of Process Parameters

Now that $\hat{p}$ was found, we assumed that the defined polynomial dependency of ϕ on *T* and *C* is valid in a neighborhood $N_{\xi }$ of the experimental conditions, which were used for the model fitting. In other words, the polynomial description is, at best, a local approximation of the underlying physical relationships and one cannot claim that this relationship is valid over the whole space of temperatures and precursor concentrations. Under this restriction, the obtained model can be used to predict the moments of the particle size distribution under arbitrary combinations of process parameters chosen from this particular neighborhood.

As introduced in the beginning, several applications require a large number of small particles in a certain period of time and various different objective functions can be defined to achieve this goal. In the given case the amount and size of particles in the mineralization solution should be appropriate over the entire time-span for homogeneous ZrbM deposition on templates. We assumed the time between preparation of solution and exposition to the substrate is 30min and the exposure time is 60min. On the one hand, the average diameter of particles during the exposure time shall be minimized while on the other hand the relative amount of ZrbM with respect to invested precursors should be maximized. For the first objective, Equation (29) is transformed to arrive at

(36) $$\begin{array}{c} Z\left(\xi ,t\right)={\left(\frac{6m_{1}\left(t,\xi ,\hat{p}\right)}{\pi m_{0}{\left(t,\xi ,\hat{p}\right)}^{3}}\right)}^{\frac{1}{3}}, \end{array}$$

the average diameter of particles. The second objective, i.e., maximizing the amount of ZrbM, is equivalent to minimizing the concentration of ${\mathrm{Zr}}^{4+}$ in mineralization solution normalized to the initial precursor concentration, leading to

(37) $$\begin{array}{c} l_{c}\left(\xi ,t\right)=\frac{c(t,\xi ,\hat{p})}{c_{0}}=1-\frac{\rho }{Mc_{0}}m_{1}\left(t,\xi ,\hat{p}\right). \end{array}$$

Note that due to the normalization by $c_{0}$, Equation (37) takes values between zero and one. To equally weigh the two optimization objectives, we thus introduce

(38) $$\begin{array}{cc} l_{Z}\left(\xi ,t\right)= & \frac{Z{\left(\xi ,t\right)}^{2}}{Z{\left(\xi ,t\right)}^{2}+Z_{d}^{2}}, \end{array}$$

with the tuning parameter $Z_{d}=1\times 10^{-4}\;mm$ and the stage cost for each measurement time point

(39) $$\begin{array}{cc} l(\xi ,t)= & l_{c}\left(\xi ,t\right)+l_{Z}\left(\xi ,t\right). \end{array}$$

It is now assured that both objectives contribute values between zero and one to the stage cost. In particular, Equation (38) is chosen as a Hill-function which takes the value 0.5 at $Z\left(\xi ,t\right)=Z_{d}$ and, thus, $Z_{d}$ can be used to tune the acceptable range of average particle diameter.

The objective function is now defined as the average of the stage cost during the previously defined time span, i.e.,

(40) $$\begin{array}{c} L\left(\xi \right)=\frac{1}{K}\sum_{30<t_{k}<90}l\left(\xi ,t_{k}\right), \end{array}$$

with *K* the number of measurements in the interval t∈[30,90], in the present case chosen as K=15.

Similarly to the parameter fitting problem, it now remains to solve the following optimization problem to determine the optimal process parameters which should lead to a large population of nanoscaled particles:

Find $\hat{\xi }=\left\{\hat{T},\hat{C}\right\}$ such that

(41) $$\begin{array}{c} L\left(\hat{\xi }\right)=\min_{\xi \in N_{\xi }}L\left(\xi \right). \end{array}$$

Due to the negative quadratic dependence of parameters *a* and *d* discussed in the previous section, we choose the neighborhood over which we search for optimal process parameters as

(42) $$\begin{array}{c} N_{\xi }\left\{\{T,C\}\;|\;25<T<60,\;20<C<50\right\} \end{array}$$

**Figure 5:**
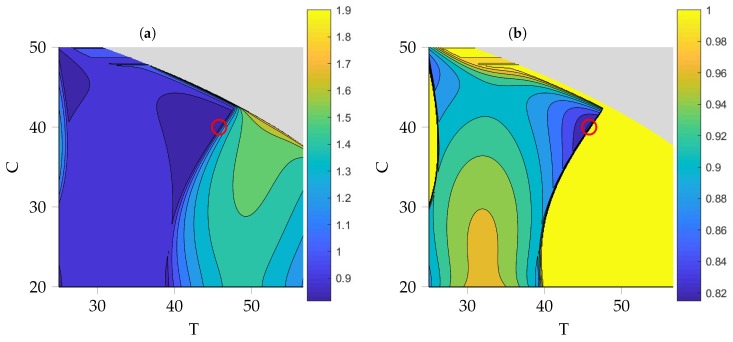
Objective function *L* over process parameters *T* and *C*. (**a**) Global view. (**b**) Local view with adapted color index to visualize differences in the area of minima. Red circle indicates minimum of L(ξ) with $\hat{T}=45.8{\;}^{\circ }C$ and $\hat{C}=40\;mM$. No value can be obtained for the process parameters in the grey area due to a nucleation rate value of d=0 and an initial condition of zero particles.

The evaluation of objective function *L* defined in Equation (40) in this neighborhood is depicted in Figure 5 with two different color indices to better visualize the differences in *L*. From Figure 5a one can distinguish between three different regions: the gray one for both *T* and *C* large, the mainly blue region with small values of *L* for small *T* and a region with high values of *L* for large *T*. These regions are mainly determined by the values of the nucleation parameter *d* and the rate of agglomeration $\beta_{1}$. In the gray region, *d* is zero and due to our choice of initial values for the moments, i.e., $m_{0}\left(0\right)=m_{1}\left(0\right)=0$, the system doesn’t evolve at all, thus, no value for *L* can be determined. The high *L* region mainly differs from the low *L* region in the value of $\beta_{1}$. As expected, as soon as agglomeration takes place, the average size of particles will increase significantly, leading to this sharp discrimination between the two areas. In Figure 5b, the color index is adjusted so that the small differences between the values for *L* around the optimum become apparent. The total minimum (red circle) is located in an area very close to the edge where agglomeration starts to happen. These results suggest a process temperature of $\hat{T}=45.8{\;}^{\circ }C$ and a precursor concentration of $\hat{C}=40\;mM$ for an optimal particle size distribution with respect to the objective function Equation (40).

**Figure 6:**
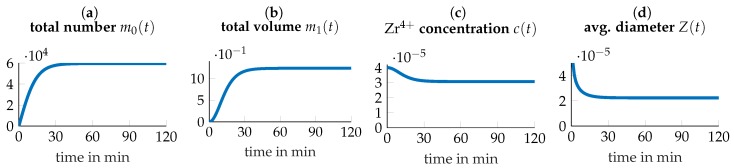
Trajectories of the system under optimized conditions $\hat{T}=45.8{\;}^{\circ }C$ and $\hat{C}=40\;mM$.

The trajectories of the simulated system at $\hat{p}$ and $\hat{\xi }$ are depicted in Figure 6 and as seen in Figure 6c, roughly 25% of precursors are mineralized. Further, shown in Figure 6d, the average diameter of particles converges to about $2.4\times 10^{-5}\;mm=24\;nm$. So, although the conversion rate of particle doesn’t seem to be very high, the amount is substantial and the average size in the desired nanometer range.

## 4. Discussion

Due to the good agreement of the model and the experimental data, we assume that the derived model realistically captures the main growth processes in the present mineralization system and conclude that this model is suitable for predicting the temporal development of the total number and total volume of particles, i.e., the zeroth and first moment of the particle size distribution, in a certain range of process parameters. We identified two regions of process parameters, one where the growth of particles is mainly driven by agglomeration and second where molecular addition of Zr-tetramers or Zr-oligomers on growing particles is the main mechanism for particle growth. Due to the non-linearities of the model, oscillations or even chaotic behavior cannot be excluded categorically. Thus, a more detailed analysis of the convergence properties of the model would be desirable to answer the question of whether and under which parameters such dynamic behaviors are possible. More elaborate strategies for obtaining an optimal particle size distribution such as a time-dependent temperature profile may be considered for future studies. By switching from a constant to a time-dependent temperature scenario one may exploit the different growth mechanisms to obtain more desirable particle size distributions. For instance a high initial temperature is beneficial for the generation of small particles by nucleation but at the same time leads to agglomeration of these particles. If the temperature is rapidly decreased after a certain time this negative effect may be circumvented. Ultimately, closing the loop and controlling the process parameters based on real time observations of the system may be studied to closely control the moments of the particle size distribution in an automatic control fashion. In a further step, limit values with regard to the pH value of the mineralization solution and the temperature can also be included, especially for bio-inspired material formation processes on an organic template. This ensures the chemical and thermal stability of the organic template.
